# Prepare Romania: study protocol for a randomized controlled trial of an intervention to promote pre-exposure prophylaxis adherence and persistence among gay, bisexual, and other men who have sex with men

**DOI:** 10.1186/s13063-024-08313-4

**Published:** 2024-07-10

**Authors:** Corina Lelutiu-Weinberger, Mircea L. Filimon, Anna M. Zavodszky, Mihai Lixandru, Lucian Hanu, Cristina Fierbinteanu, Raluca Patrascu, Adrian Streinu-Cercel, Sergiu Luculescu, Maria Bora, Irina Filipescu, Cristian Jianu, Lisa B. Hightow-Weidman, Aimee Rochelle, Brian Yi, Nickie Buckner, Sarit A. Golub, Ilana Seager van Dyk, Julian Burger, Fan Li, John E. Pachankis

**Affiliations:** 1 School of Nursing, Columbia University, 560 West 168th Street, New York, NY 10032 USA; 2 The Romanian Association Against AIDS, Bulevardul Eroilor Sanitari 49, 050471 Bucharest, Romania; 3 The National Institute of Infectious Diseases “Professor Dr. Matei Bals”, Strada Doctor Calistrat Grozovici 1, 021105 Bucharest, Romania; 4 The Clinical Hospital of Infectious Diseases, Str. Iuliu Moldovan, nr. 23, 400000 Cluj-Napoca, Romania; 5 College of Nursing, Florida State University, 98 Varsity Way, Tallahassee, FL 32305 USA; 6 One Cow Standing, 300 W Morgan St Ste 1425, Durham, NC 27701 USA; 7 Department of Psychology, Hunter College of the City University of New York, 695 Park Avenue, New York, 10065 NY USA; 8 School of Psychology, Massey University, PO Box 756, Wellington, 6140 New Zealand; 9 Department of Social and Behavioral Sciences, Yale School of Public Health, 60 College Street, New Haven, CT 06610 USA; 10 Department of Biostatistics, Yale School of Public Health, 60 College Street, New Haven, CT 06520 USA; 11 Social and Behavioral Sciences, Yale School of Public Health, 135 College Street, New Haven, 06520 CT USA

**Keywords:** Gay, bisexual, and other men who have sex with men (GBMSM), HIV/AIDS, Mental health, Mobile health (mHealth), Pre-exposure prophylaxis (PrEP), Stigma

## Abstract

**Background:**

Gay, bisexual, and other men who have sex with men (GBMSM) represent a high-risk group for HIV transmission in Romania, yet they possess few resources for prevention. Despite having no formal access to pre-exposure prophylaxis (PrEP) through the health system, GBMSM in Romania demonstrate a high need for and interest in this medication. In anticipation of a national rollout of PrEP, this study tests the efficacy of a novel strategy, Prepare Romania, that combines two evidence-based PrEP promotion interventions for GBMSM living in Romania.

**Methods:**

This study uses a randomized controlled trial design to examine whether GBMSM living in Romania receiving Prepare Romania, a culturally adapted counseling and mobile health intervention (expected *n* = 60), demonstrate greater PrEP adherence and persistence than those assigned to a PrEP education control arm (expected *n* = 60). Participants from two main cities in Romania are prescribed PrEP and followed-up at 3 and 6 months post-randomization. PrEP adherence data are obtained through weekly self-report surveys and dried blood spot testing at follow-up visits. Potential mediators (e.g., PrEP use motivation) of intervention efficacy are also assessed. Furthermore, Prepare Romania’s implementation (e.g., proportion of enrolled participants attending medical visits, intervention experience) will be examined through interviews with participants, study implementers, and healthcare officials.

**Discussion:**

The knowledge gained from this study will be utilized for further refinement and scale-up of Prepare Romania for a future multi-city effectiveness trial. By studying the efficacy of tools to support PrEP adherence and persistence, this research has the potential to lay the groundwork for PrEP rollout in Romania and similar contexts.

Trial registration

This study was registered on ClinicalTrials.gov, identifier NCT05323123, on March 25, 2022.

## Background

Gay, bisexual, and other men who have sex with men (GBMSM) remain an under-prioritized yet high-risk group with regard to HIV/AIDS prevention in the Central-Eastern European (CEE) country of Romania [1]. The World Health Organization (WHO) reports that out of 15 countries in its Central European region, Romania had the second-highest number of new HIV cases transmitted by sex between men in 2021. Romania also had the fourth highest HIV incidence in 2021 out of all 31 European Union/European Economic Area (EU/EEA) countries. Furthermore, while the annual incidence of HIV cases among GBMSM has been declining in most EU/EEA countries since 2015, cases among this group appear to have increased in Romania [2]. In 2022, 30% of new HIV cases in Romania were attributable to sexual contact between men (up from 4% in 2007), and another 43% of cases occurred in men reporting heterosexual contact [3]. However, due to low levels of “outness” among GBMSM as well as homophobic stigma and discrimination in Romania, many HIV cases among GBMSM may be misattributed to heterosexual transmission [4, 5]. Furthermore, a bio-behavioral survey of GBMSM in six major Southern and Eastern European cities found that Bucharest (Romania’s capital) had the highest proportion of undiagnosed HIV infections [6]. Yet, 2023 is the first year in which national government funds have been allocated for HIV/STI prevention among GBMSM in Romania [7, 8].

Romania has some of the highest levels of homophobic attitudes and lesbian, gay, bisexual, transgender, and other identities (LGBTQ+) discrimination in Europe. While Romania’s entry into the EU spurred some increases in LGBTQ+ legal rights, GBMSM remain highly stigmatized and most continue to conceal their identities due to experienced and anticipated stigma [9–11]. Homophobic stigma serves as a principal HIV risk factor that keeps GBMSM living in Romania both from accessing HIV prevention services and “hidden” from official HIV and sexuality transmitted infection (STI) surveillance [1, 5, 12]. For example, increasing reports indicate that cases of HIV transmitted via sex between men are frequently underreported and/or misclassified as heterosexual [1, 13]. Untreated STIs also represent a primary risk factor for HIV transmission [14]; yet, comprehensive, appropriate STI screening for this population in Romania is rare [4, 15].

These trends make it clear that the time has come to prepare for the national rollout of biomedical HIV prevention, namely pre-exposure prophylaxis (PrEP), in Romania. Daily, orally administered tenofovir/emtricitabine (TDF/FTC), the most common form of PrEP, has demonstrated high efficacy in preventing HIV infection among GBMSM, with HIV risk reductions as large as 96% with high adherence (i.e., at least four doses per week) [16–18]. GBMSM living in Romania would benefit from PrEP use given their high HIV rates [2, 4] and interest in PrEP [19]. Still, although increasing numbers of GBMSM living in Romania are demanding PrEP [19], they have no formal way to access it, which forces many to purchase PrEP online and use it without medical guidance or monitoring [20]. Such behaviors have the potential to pose both individual (e.g., renal toxicity) and population-level (e.g., increased drug resistance) health risks, placing further urgency on systematic PrEP rollout. A 2017 survey of GBMSM living in Romania indicates their low access to and high unmet need for PrEP, with only 1% of participants having ever taken it [4, 19].

Mobile health (mHealth) technology constitutes an ideal HIV-prevention strategy for GBMSM, including those living in Romania, given its accessibility and safety in stigmatizing locales. Over a decade of research has established the willingness of GBMSM, including in CEE, to use mobile devices (e.g., phones, tablets, laptops) for HIV- and other health-related promotion [21–29]. Mobile tools are the primary means for GBMSM living in Romania to form and navigate social and sexual networks, especially given high stigma-driven concealment demands [9], and are therefore ideal intervention platforms [30]. Romania also possesses a high rate of smartphone penetration with 90% of young adults owning a smartphone [31] and 9 in 10 Romanians using mobile phones daily [32].

Motivated by the rapidly increasing HIV incidence among GBMSM living in Romania, a lack of national healthcare system preparedness, and the instrumentality of mHealth in reaching this key population, this study examines the efficacy of Prepare Romania, a combination of two efficacious United States (U.S.)-based PrEP education and adherence tools: (1) Sparking PrEP Awareness Research and Knowledge (SPARK) [33, 34] and (2) HealthMpowerment (HMP) [24, 35, 36]. In Prepare Romania, SPARK, an in-person motivational interviewing counseling intervention [34], is offered to GBMSM living in Romania at risk for HIV together with the HMP application (“the app”), an educational and medication adherence support mobile app [24, 35]. To create Prepare Romania, we culturally adapted, integrated, and tested these tools for GBMSM living in Romania through two pilot trials conducted between September 2020 and March 2022 [37]. Pilot trials showed that the SPARK protocol was acceptable as originally configured, while the HMP app underwent several modifications to create the Prepare Romania app, such as the addition of trackers desired by participants (e.g., smoking, spending habits), contextualized educational resources, a revised color scheme, and various avatar styles [37]. All procedures were pilot-tested and refined with 10 participants in the first pilot (1-month follow-up) and 20 participants in the second pilot (3-month follow-up) [37].

The aim of the current trial is to determine if PrEP adherence and persistence is significantly higher among GBMSM randomized to receive Prepare Romania compared to those randomized to receive a PrEP education-only treatment across two Romanian cities. Additionally, the study explores implementation determinants to identify barriers to and facilitators of implementing the Prepare Romania protocol at an individual and institutional level. Proctor and colleagues’ [38] conceptual framework will be applied in the analysis of the intervention’s implementation via interviews conducted with the counselors, physicians, clinic administrators (e.g., medical directors), local implementing staff, and participants involved in the study. Results may inform a future effectiveness-implementation hybrid trial of this intervention across Romania and similar countries in CEE.

## Methods

### Recruitment

Recruitment and enrollment for the current trial began in April 2023 and include GBMSM living in or within 40 miles of Romania’s capital, Bucharest, and another major city, Cluj-Napoca. As a standard of practice, Asociaţia Română Anti-SIDA (ARAS; Romanian Association Against AIDS), the study’s implementation partner, provides weekly free and confidential HIV/STI testing at “Checkpoint” venues in each of the study cities. GBMSM seeking testing services at ARAS “Checkpoints” are referred to the study if they receive a negative result on a rapid HIV test. Interested participants complete a five minute online screener, followed by the informed consent process, if found eligible. Recruitment will conclude when 60 participants from each city have been enrolled, found eligible, and started the PrEP regimen.

### Eligibility

Participants’ eligibility is determined based on data from their screener form (see criteria 1–6 below) and their baseline medical visit (see criteria 7–8 below). Participants are eligible if they (1) were assigned male sex at birth and have a current male gender identity; (2) are at least 18 years old (as Romanian law prohibits prescribing medication to minors without parental consent, which may jeopardize individuals’ wellbeing given pervasive homophobic stigma in Romania); (3) have had, in the last 6 months, at least one act of condomless anal sex with an HIV-positive or status-unknown male partner and/or a diagnosis of a bacterial STI; (4) receive a preliminary-negative rapid HIV test result at a “Checkpoint” venue; (5) plan to live in Romania for next 6 months; and (6) own a mobile device (e.g., smartphone, tablet, laptop) for usage of the Prepare Romania app. At the baseline medical visit, eligibility-related testing includes (7) confirmation of HIV-negative status via blood draw and (8) renal function testing. Participants are excluded if they declare (1) enrollment in a concurrent HIV prevention study conducted by our team; (2) past-year psychiatric hospitalization, psychotic and manic symptoms, or current mood-stabilizing or antipsychotic medication prescription; (3) serious medical condition(s) or evidence of health issues (e.g., poor renal function) identified by study’s baseline biological testing; or (4) any other condition that, in the investigators’ judgment, interferes with safe study participation.

### Design

**Fig. 1:**
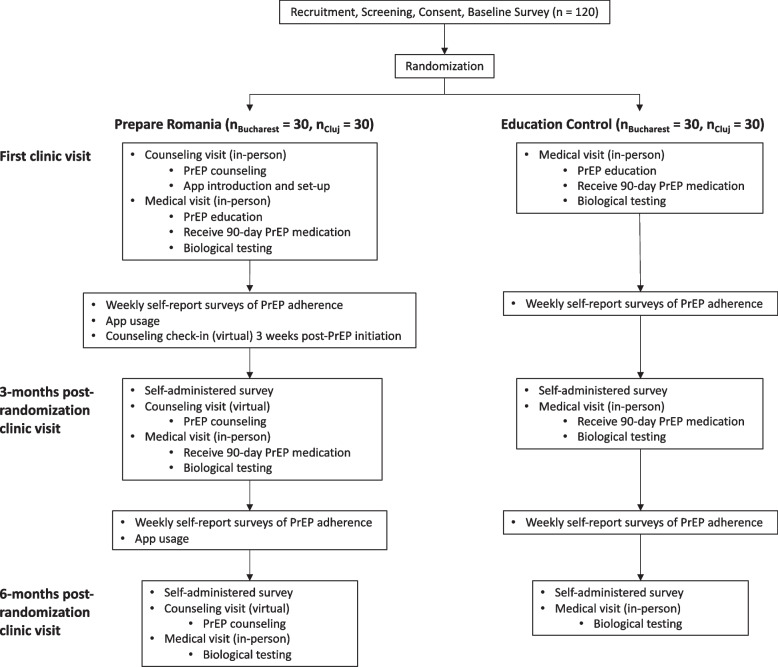
Participant timeline

**Fig. 2:**
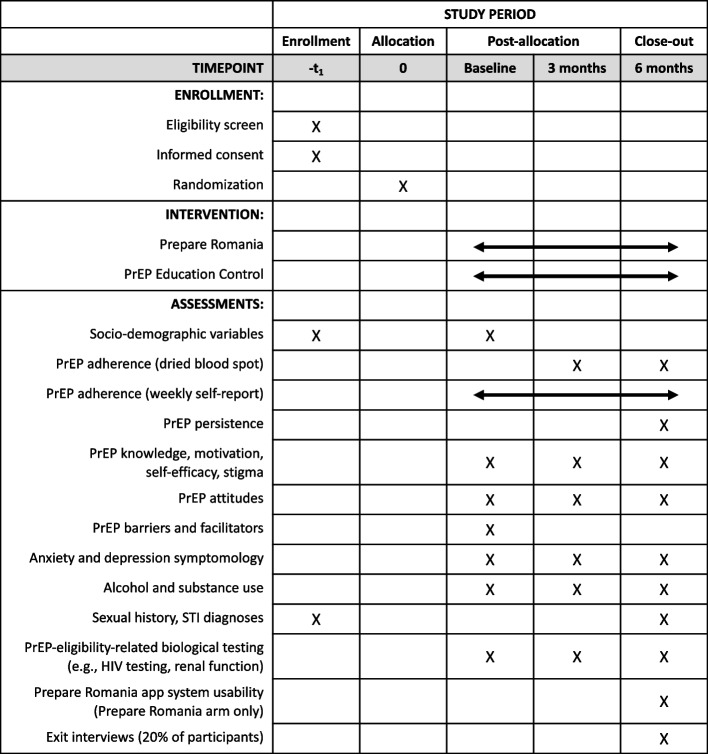
SPIRIT figure summarizing schedule of enrollment, interventions, and assessments

The study employs an individually randomized, unmasked randomized controlled trial (RCT) to compare outcomes between participants in the Prepare Romania intervention arm and those in the PrEP education control (EC) arm. Participants randomized to Prepare Romania are hypothesized to achieve significantly higher PrEP adherence and persistence rates than participants randomized to the control arm. Both primary and secondary outcomes will be measured at baseline as well as at 3 and 6 months post-randomization, as described below in the “Study assessments and measures” section. Figure 1 illustrates participant flow by study arm, and Fig. 2 presents the study timeline in the form of a SPIRIT figure.

### Randomization

Upon completing the informed consent and initial screening processes, participants are randomized to either the intervention or control arm. Randomization is conducted by the study coordinator using a computer program that produces a 1:1 allocation ratio. While the target sample size is 60 participants per city, randomization must occur before the first medical visit for practical reasons. Therefore, we have programmed randomization sequences of 70 participants per city to allow up to 10 participants per city to be excluded based on the results of the first medical visit (e.g., participants deciding not to start PrEP or PrEP being contraindicated based on biological testing, as below). Upon randomization, an email is sent to the participant as well as research staff, who inform participants of next steps.

### Clinic visits

The two study sites are the National Institute of Infectious Diseases (NIID) in Bucharest and the Regional Center of Evidence and Monitoring of HIV Infection Cluj-Napoca (CREM Cluj). For the purposes of the current study, each study site has been established with a team comprising of both a psychologist serving as the PrEP counselor and an infectious disease physician. After randomization, participants assigned to the Prepare Romania arm meet with the PrEP counselor, followed by a medical visit with the infectious disease physician. Participants randomized to the control arm only undergo the medical visit and do not receive PrEP counseling. All participants complete follow-up visits at 3 and 6 months post-randomization mirroring those at baseline. Participants receive 90 days of PrEP medication at the baseline and 3-month medical visits. At the 6-month follow-up, participants are provided with a list of verified PrEP sources (e.g., online pharmacies outside of Romania, a Bucharest-based pharmacy that recently began carrying PrEP), should they decide to continue taking PrEP.

### Participant retention

The study coordinator and local implementing staff from ARAS monitor patient progress (e.g., appointment attendance, survey completion) using REDCap. The study coordinator ensures timely documentation of events (e.g., appointment dates and attendance, test results) by local staff. Participants also provide their phone number and email address so that local staff can contact them with reminders about completing study appointments, weekly PrEP adherence surveys, and follow-up assessments, as needed. Participants receive monetary compensation: 120 RON after completing all baseline study components, 140 RON after completing the 3-month follow-up, 160 RON after the 6-month follow-up, and 120 RON for completing an exit interview if selected. These amounts were determined in consultation with the Romanian team to avoid potential coercion.

### Condition 1: Prepare Romania

Prepare Romania combines and adapts two previously developed PrEP-related programs: (1) SPARK, a counseling intervention originally developed in the U.S. to be delivered in-person [34], and (2) HMP, an app-based tool intended to support medication adherence through customized features such as behavioral tracking and educational resources [24]. These behavioral interventions are delivered before the medical visit in which a physician discusses and prescribes PrEP. Through stakeholder consultations, working groups, and pilot testing, the components of SPARK and HMP have been previously adapted and refined to increase cultural appropriateness, feasibility, and acceptability for GBMSM living in Romania [37]. The counseling visits, based on SPARK, utilize motivational interviewing techniques to promote participants’ sexual health and HIV prevention through a “sex-positive” approach as well as by emphasizing positive impacts of sexual-health-promoting behaviors as opposed to potential hazards of sexual risk behaviors [39].

In the Prepare Romania condition, a PrEP counseling session occurs before the medical visit to support PrEP adherence and persistence by providing a rationale for daily dosing (e.g., protective drug concentrations), developing plans to integrate PrEP use into participants’ daily routines (e.g., when, where, how), assisting with side effect management (e.g., over-the-counter remedies for nausea), discussing disclosure of PrEP use to avoid stigma (e.g., when and whom to tell), and anticipating potential barriers to PrEP adherence (e.g., traveling).

At their baseline counseling visit, participants are introduced and granted access to the app, through which they can access various educational resources and social networking features related to PrEP as well as other sexual, physical, and mental health topics. These resources and features include informational articles and activities, badges for app use, communication with other participants via an in-app forum, and tracking of daily PrEP use, as well as tracking of other behaviors and experiences found to be important by GBMSM who participated in the intervention’s adaptation research (e.g., cigarette smoking, alcohol consumption, spending habits). Participants meet with a nurse to complete a blood draw that confirms their HIV-negative status and verifies renal function. Participants are told not to begin using PrEP until the physician confirms with them by phone that PrEP is indicated for them based on their test results. Finally, 3 weeks after PrEP initiation, participants complete a brief, virtual check-in with the counselor to discuss any issues or concerns they may have with medication adherence or app usage.

All participants regardless of condition then complete a medical visit with an infectious disease physician. The physicians conducting these medical visits receive training in efficacious PrEP messaging [40]. During the visits, physicians first provide basic PrEP information: what it is (e.g., definition), how it works (e.g., prevents viral replication), efficacy (e.g., clinical trial evidence), and side effects (e.g., type, duration), as well as the importance of daily use (e.g., to build and sustain protective medication levels) and continued condom use (e.g., PrEP is an added layer of protection against HIV but not other STIs). Physicians answer questions, provide an informational brochure, order laboratory tests to ensure that PrEP use is indicated, and provide a prescription for a 90-day supply of PrEP disbursed at the clinic. Participants in the Prepare Romania condition undergo the counseling and medical visit at baseline as well as at 3 and 6 months post-randomization.

### Condition 2: PrEP education control (EC)

A PrEP EC was selected based on the low level of PrEP knowledge among GBMSM living in Romania [4], who would likely benefit from a comprehensive educational intervention. Participants in this condition undergo the above medical visit, but not the counseling session, at baseline as well as at 3 and 6 months post-randomization. Participants in this arm undergo the same biological tests and receive PrEP as described above for the Prepare Romania condition.

### Biological testing

#### HIV testing

All participants are initially tested at the “Checkpoint” venue using a rapid HIV test. A confirmatory HIV test is completed following the medical visit via a blood draw at the clinic’s laboratories.

#### PrEP-specific testing

Creatinine, hepatitis B surface antigen, and hepatitis C antibody testing is performed at each visit, as part of standard-of-care for PrEP use. Upon review of lab results by physicians, participants may begin PrEP use.

#### Dried blood spot (DBS) testing

DBS testing is performed at the clinic at each follow-up medical visit to test for PrEP metabolites. DBS cards are air-dried, packaged with desiccant, and stored at − 20 °C until bulk shipment is conducted through a secure courier company to the University of North Carolina Clinical Pharmacology and Analytical Chemistry Laboratory. Adherent PrEP use will be determined via blood plasma concentration indicating four or more doses taken per week (tenofovir-diphosphate (TFVdp) concentration ≥ 1000 fmol).

### Training and fidelity monitoring of PrEP counselors and physicians

Two master’s-level psychologists to serve as PrEP counselors and two infectious disease physicians (one from each city) were identified through ARAS, based on prior collaborative relationships and their GBMSM-affirmative practices. The research team met with the counselors and physicians before the trial to establish study protocols and provide training in SPARK for approximately 10 hours [37]. The counselors and physicians had prior knowledge of PrEP, which the training reinforced by reviewing evidence of its safety profile and efficacy, the importance of biological testing for establishing eligibility, PrEP dosing considerations, PrEP side effects, and adherence concerns. The clinical psychologist investigator of this study who co-developed SPARK in the U.S. provided comprehensive training to the PrEP counselors in motivational interviewing for adherence-related behavioral skills, the core techniques of SPARK, and thus Prepare Romania. The counselors were trained on the SPARK protocol and practiced delivering the intervention in mock sessions until fidelity was reached based on study team review. The delivery of Prepare Romania to each participant is audio-recorded, and 20% of counseling sessions are transcribed, translated, and reviewed for fidelity. Treatment fidelity monitoring occurs using checklists based on the manualized intervention. A clinical psychologist on the team reviews counseling transcripts and provides feedback to the PrEP counselors during biweekly remote, one-hour group supervision sessions, as in prior studies [30, 41, 42]. Counselors were also trained in the use of the app and to troubleshoot potential technical issues with the participant, with support from the research team as needed.

### Study assessments and measures

All participants complete the same online self-assessments at baseline (after completing the screening and consent process, but before being randomized and attending baseline visits) and at 3 and 6 months post-randomization (before attending the corresponding follow-up visits). All measures have been validated with GBMSM living in Romania (*n* = 30) during the above-mentioned formative, pilot phases of this project [37].

#### Primary outcome

PrEP adherence will be measured via biological (primary) and self-report (secondary) measures. To collect the primary outcome, DBS sample collection occurs at each follow-up visit, allowing for analysis of binary (≥ 1000 fmol vs. < 1000 fmol/punch TFVdp) and continuous (fmol TFVdp) operationalizations of the adherence outcome. PrEP adherence of ≥ 4 doses per week, equivalent to ≥ 1000 fmol TFVdp, has been shown to be protective against HIV transmission [43]. The binary outcome will serve as the primary outcome.

#### Secondary outcomes

The secondary PrEP adherence outcome relies on participant self-report. Once participants receive the first 3-month supply of PrEP medication, they begin receiving weekly links to an online PrEP tracker survey to self-report daily adherence until the completion of the study. There, participants mark the days over the past week on which they took or missed PrEP. Each participant’s weekly survey results will be averaged to determine their mean number of PrEP doses taken per week over the past 3 months. In other words, for the 3-month endpoint, surveys from baseline to the 3-month mark will be considered, and for the 6-month endpoint, surveys from the 3- to 6-month marks will be utilized. This secondary PrEP adherence outcome will be analyzed as both a continuous (average number of PrEP doses taken per week when data were reported, from 0 to 7) and binary variable (0–3 doses per week, on average vs. ≥ 4 doses per week, on average).

Other secondary outcomes include PrEP persistence (i.e., continuing vs. stopping without restarting PrEP for the 6-month study period, measured dichotomously); PrEP knowledge, motivation and self-efficacy [44]; PrEP attitudes [45]; PrEP barriers and facilitators [46]; PrEP stigma [47]; anxiety and depression symptomology reported using the Generalized Anxiety Disorder 7-item (GAD-7) [48] and Patient Health Questionnaire-9 item (PHQ-9) [49], respectively; degree of “outness” [50]; and alcohol and substance use (reported using the Alcohol, Smoking and Substance Involvement Screening Test (ASSIST)) [51]. At the time of screening and the 6-month follow-up survey, participants are also asked about their number of acts of condomless anal sex with an HIV-positive or status-unknown male partner over the past 6 months and whether they have received a diagnosis of a bacterial STI in the past 6 months.

At baseline, participants also indicate their age, gender, ethnicity, sexual orientation and related developmental milestones (e.g., age of realizing they are LGBTQ+) [52], relationship status, education level, residential urbanicity, and level of religiosity when growing up.

#### Implementation outcomes

We will review intervention data (e.g., enrollment rates, retention at 3- and 6-month follow-ups, as well as app analytics including frequency and types of app interaction, counselor and physician qualitative feedback on intervention delivery experiences, participant System Usability Scale scores [53]) and counselor fidelity in intervention delivery as described above. We will also interview 24 participants (20% of the sample) with various levels of engagement, both who continued and discontinued PrEP; the study counselors, physicians, and local implementing staff; the directors of the two study clinics; and two Ministry of Health members. Participants will be asked about their experiences taking PrEP (e.g., adherence facilitators and barriers) and participating in the study (e.g., what went well, what could be changed or improved). The local coordinators, counselors, and physicians will be asked about feasibility of integrating Prepare Romania into their practices. Two physicians from other Romanian cities not involved in this trial will be interviewed about potential implementation of Prepare Romania in their clinics. The two clinic directors will be asked about Prepare Romania’s compatibility with their practice model and integration into standard of care. Finally, two Ministry of Health members will be interviewed about integrating Prepare Romania within the Ministry’s strategic plan for HIV/AIDS prevention and care [8].

### Data management

All databases and files are stored on one of the principal investigator’s (PI’s) secure institutional servers, while all data are marked with participant study identification numbers (study IDs), which are not directly connected to any personally identifying information. All participant tracking data (e.g., enrollment, retention, follow-up schedules, study visit completion) are entered by project staff into the secure and Health Insurance Portability and Accountability Act (HIPAA)-compliant REDCap system housed at the same PI’s institution. All participant assessment data is self-administered via HIPAA-compliant Qualtrics links. The resulting files are downloaded onto one of the PI’s institutional servers and are password protected. DBS samples are collected by the study physicians and stored, as described above, under participants’ study IDs. The study coordinator monitors and checks data for completion and accuracy on a weekly basis.

### Power considerations

As the scope of this study involves estimating the rates of adherence for study participants to inform a future effectiveness-implementation hybrid trial, a formal sample size calculation was not performed. However, we followed the recommendations of Whitehead and colleagues [54] to determine the sample size of this pilot trial. In a previous RCT that evaluated the SPARK intervention against a control condition where only PrEP education was provided (similar to the current trial), 94% of the intervention and 85% of the control arm demonstrated protective PrEP levels at the 3-month follow-up point, while 92% of the intervention and 86% of the control arm did so at the 6-month follow-up [39, 55]. Therefore, a conservative effect size in terms of rate difference is estimated as 6%, with a pooled standard deviation of 0.32. This provides a standardized effect size of 0.19. Whitehead and colleagues [54] suggest, as a rule of thumb, including at least 25 participants in each condition for a pilot trial if the standardized effect size is between 0.1 and 0.3. Therefore, we plan to enroll 60 participants in each arm, such that the number of participants per arm retained in the study at the 6-month timepoint exceeds 25 after accounting for attrition. This is a conservative sample size estimate, as retention for PrEP users across 12 months in the original SPARK trial was as high as 89% (or 11% attrition) [55].

### Data analyses

#### Aim 1: Test the preliminary efficacy of Prepare Romania, a combined counseling and mHealth intervention designed to promote PrEP adherence and persistence among GBMSM living in Romania, through an RCT

Across the two study conditions, differences will be explored in the primary outcome of PrEP adherence (measured via DBS) as a binary variable, as well as the secondary outcomes, such as PrEP adherence (measured via DBS) as a continuous variable, PrEP adherence measured via self-report (both as binary and continuous variables), PrEP persistence, PrEP-related self-efficacy, and mental health at 3 and 6 months post-randomization. We hypothesize that compared to those receiving the PrEP EC condition, individuals receiving the Prepare Romania intervention will demonstrate greater PrEP adherence, persistence, and self-efficacy, as well as improved mental health.

Data across the two cities will be aggregated to ensure sufficient sample size. Descriptive statistics will be computed to describe the trial based on baseline covariates (e.g., PrEP motivation), PrEP adherence (DBS and self-reported measures), and secondary outcomes (e.g., PrEP persistence, PrEP-related self-efficacy, mental health) at 3 and 6 months post-randomization. Categorical variables will be summarized by condition using proportions and frequencies and continuous variables with means and standard deviations (or with medians and interquartile ranges for skewed outcomes). Missing data will be reported, and reasons for missingness will be investigated and recorded. For each follow-up timepoint, exploratory analyses based on the Pearson/Spearman correlations will be performed regarding the outcome variables and study condition to determine the association and trend of any changes in outcome by condition.

The primary analysis will compare the proportion of participants with protective levels of TFVdp at 3 and 6 months post-randomization between the study conditions using generalized linear mixed models accounting for within-participant correlation over time. The statistical model will provide estimates of the percentages of participants with protective levels of TFVdp (plus 95% confidence intervals) by study arm and assessment point and test for the interaction between time and condition to determine whether adherence in study conditions changed over time. Secondary outcomes will be analyzed analogously. Each model will include the stratification factor (i.e., city) and several baseline covariates that are considered to be predictive of the outcomes and associated with potential attrition, to potentially improve precision and address outcomes missing seemingly at random [56, 57]. Intent-to-treat analyses will be utilized for the aforementioned models.

#### Aim 2: Assess implementation of Prepare Romania

We will identify individual and institutional barriers and facilitators of implementing Prepare Romania [38, 58, 59] via interviews. We will use Proctor and colleagues’ [38] framework to guide feasibility, acceptability, appropriateness, fidelity, and sustainability analyses. The interviewed participant sample size (*n* = 24) was estimated to meet thematic saturation [60, 61]. Interviews will be audio-recorded, transcribed, and translated into English. Transcripts will be independently coded by two trained coders, as in previous intervention adaptations [30, 41, 62], using thematic coding [63, 64]. Data will be indexed via *open coding* to identify new participant themes and* axial coding* guided by intervention targets (e.g., PrEP adherence) as well as facilitators and barriers to implementation.

### Data and safety monitoring

This study was not determined to be of greater than minimal risk, based on the investigative team’s experiences with conducting similar trials in the U.S. and Romania [37]. Any unexpected or serious adverse events that occur during the course of the study will be reported by the PIs to the Committee on Human Research at Columbia University and the Romanian NIID, in accordance with current guidelines for reporting adverse events. Furthermore, the Romanian and U.S. study teams meet biweekly, or more often if needed, to monitor study progress. Adverse events (AEs) include symptoms reported by the participant and abnormal measures of clinical importance evaluated by Drs. Pachankis (clinical psychologist) and Hightow-Weidman (infectious disease physician). The investigative team will decide whether the AE could be related to study participation. During the pilot study for this trial, there were no noted side effects or adverse events.

All serious or unexpected adverse events that are definitely, probably, or possibly related to study participation will be reported to the Columbia University within 48 hours of site awareness. The U.S. National Institutes of Health (NIH) project officer will be provided copies of these reports and informed of any actions taken by the Committee on Human Research as a result of such events. The PIs will promptly inform the NIH and Columbia University’s Committee on Human Research of any changes in the protocol relevant to safety as the study is being performed. They will additionally notify NIH of any actions taken by the Committee on Human Research during continuing study review and of any major changes in the status of the ongoing protocol.

The team has assembled a three-member Data and Safety Monitoring Board (DSMB), who are independent of the research team, study, and sponsor, to determine safe and effective conduct and recommend conclusion of the study if significant risks develop or if the trial is unlikely to be concluded successfully. The DSMB members have expertise in PrEP for GBMSM, CEE, biostatistics, and clinical trials. The first DSMB meeting was held prior to participant enrollment and will be held annually or more often if needed. During these meetings, the DSMB members review study design and procedures and plan for monitoring study data, and creating interim outcomes may be advised. A report is generated after each DSMB meeting, which is submitted to the NIH with each overall study progress report. Protocol modifications (e.g., eligibility criteria, outcomes) will be reported to the DSMB, ethics committees, and the sponsor when relevant.

### Potential risks

Potential risks consist of (1) discomfort or distress arising from completing the screener, assessments, or biological testing; (2) breaches of privacy and confidentiality; and (3) PrEP side effects. Based on the team’s experience conducting PrEP-related studies, protocols have been developed for recruitment, consent, assessments, biological testing, and intervention delivery. Prior to any contact with participants or data, all staff complete the required research ethics certifications and training in issues pertinent to research among GBMSM in an international context.

### Minimizing participant distress during assessments and biological testing

It is possible that participants may experience some discomfort in responding to screening and assessment questions, biological testing, as well as discussions regarding sexuality and sexual experiences during the counseling sessions. Sexual behavior among men and its associated stigma are sensitive topics that may elicit discomfort. Biological testing and receipt of HIV-positive results may induce distress.

While every possible step will be taken to minimize such risk, if participants have any concerns about any aspect of the study, consent documentation specifies that they may refuse to continue with the study at any time without penalty. Participants are also made aware that they do not have to respond to any questions which they do not wish to address. The two PrEP counselors have been trained in GBMSM-affirmative clinical practice by one of the study PIs, a clinical psychologist. They also possess advanced degrees in a mental health field (e.g., psychology) and have employed GBMSM-affirmative HIV-related care in their practice prior to and during this study. Under the supervision of the clinical psychologist PI, one of counselors is available to meet with study participants to discuss study-related distress and to refer participants to counseling services regardless of ability to pay. To address suicidal ideation, the self-administered surveys are programmed to send an instant message to the team whenever participants report having thought that they would be better off dead or of hurting themselves in the prior 2 weeks [49]. Study staff contact the participant by phone to perform a safety assessment and determine whether referral or immediate linkage to care is needed.

All participants receive biological testing as described above during the in-office medical visits (at baseline and 3 and 6 months post-randomization). During consent, participants are informed of associated risks (which have been deemed minimal), including bruising around the lancet needle site and the risk of infection at the needle site. Appropriate risk-reduction counseling is provided to all participants at the time of testing. The study-associated physicians discuss HIV test results with participants as soon as they become available. Additionally, participants who receive an HIV-positive test result receive immediate counseling and medical care from study-affiliated physicians.

### Minimizing risks for potential breaches of confidentiality

It is possible that participant personal data could be breached during the course of the study, via databases, app, biological testing samples, or during counseling and medical visits sessions. The study protocols outline several safeguards to protect against this risk.

Upon consent, participants are assigned a numeric identification for use in all study-related procedures (e.g., data collection, sample storage), as described in previous sections. Any records with identifying information are stored electronically and separately from study data under password and firewall protection. All PrEP counseling and medical visits occur in private spaces.

The app is not public and is only accessible to study staff, software developers, and participants. Participants create unique log-ins (e.g., participant-chosen username and password) and are asked not to share identifying information on the app. Any identifying or inappropriate information is immediately removed by staff. Privacy-related feedback (e.g., omitting “PrEP” from push notifications and app icon) received during formative phases has been incorporated into the app. App communications are secured via industry standard encrypted secure socket layer communications links. Participants’ profiles, avatars, and usernames contain no personal information.

No presentations or publications will identify study participants individually. The exceptions to confidentiality are those defined by law and include suspicion of child abuse, elder abuse, and threat of imminent action on suicidal or homicidal ideation. Participants are informed of the exceptions to confidentiality during the consent process. The involved Committees on Human Research and NIH representatives have limited access to records (e.g., the Committee on Human Research may request a review of the associated chart). At the study’s conclusion, the link between participants’ names and data (via separate coded ID kept under password and firewall protection at Columbia University) will be destroyed. Finally, federal certificates of confidentiality do not apply to foreign countries.

### Minimizing risks related to PrEP side effects

PrEP medication for this study is composed of tenofovir and emtricitabine (TDF/FTC), which are oral antiretrovirals used originally in the treatment of HIV infection and approved by the U.S. Food and Drug Administration (FDA) for HIV prevention [65]. One potential risk of this study is experiencing known PrEP side effects, potentially including nausea, diarrhea, vomiting, flatulence, rash, rare but serious renal impairment, hypersensitivity reaction, increase in bone metabolism leading to osteopenia, and liver abnormalities. PrEP for this study is a generic form of TDF/FTC obtained through our two clinics’ pharmacies. A review of overall literature on TDF/FTC reveals an excellent safety profile. As part of the consent process, participants are advised on the potential risks associated with taking PrEP. Across several PrEP trials (ATN 082, iPrEx, and U.S. Safety Study), PrEP was shown to have few serious side effects and has no known abuse potential [66]. There is also no indication that this PrEP formulation is associated with increased rates of depression, suicidal ideation, or risk of suicide. Finally, to minimize adverse events related to TDF/FTC, individuals with serious medical conditions, kidney problems, or hepatitis B surface antigen positivity are excluded from study participation.

Participants who fill PrEP prescriptions through the pharmacies affiliated with this study are monitored for safety every 3 months over the course of their study participation. The physicians affiliated with this study will address over-the-counter remedy options in case mild side effects are experienced (e.g., nausea). PrEP will be discontinued immediately if there is evidence of toxicity related to study medication. Any needed treatment will be available through the clinic sites (affiliated with university hospitals that include all specialties).

### Auditing

Trial conduct is audited on a yearly basis during continuing review of ethics approval by the Committees on Human Research and evaluation of study progress by the study sponsor. Progress reports are submitted to each entity for their review, and questions are answered and amendments are made by the investigators as requested by the ethics committees and/or study sponsor, as applicable.

## Discussion

In December 2022, the Romanian government adopted a national strategy for addressing HIV/AIDS that documents plans to, among other activities, begin rolling out PrEP to 150 individuals per year by 2024 and dedicate annual government funding to increasing access to HIV prevention services for GBMSM [8]. This suggests a high potential for sustainability of the PrEP program tested in this study. Prepare Romania culturally adapts a combined counseling and mHealth PrEP education and adherence program for GBMSM living in Romania in response to limited availability of GBMSM-appropriate healthcare. This study lays the groundwork for swift integration of PrEP into local clinical practice using evidence-based guidance to promote PrEP adherence and persistence. Pending results of this trial, future research can continue to explore methods for ensuring that PrEP reaches those who most need it within Romania and across the region and that its implementation is facilitated by guidance from affected populations to ensure adherence.

## Trial status

Recruitment for this study began on April 4, 2023, and concluded on February 5, 2024. The anticipated last patient/last visit date is October 5, 2024 (protocol version: 1; date submitted: April 1, 2024). This manuscript was submitted to another journal several months before recruitment was complete; however, a rejection (due to the protocol being deemed as “too clinical” for that journal) was only received after recruitment was completed, preventing its submission to *Trials* before completion of recruitment.

## Data Availability

Dissemination of study findings will occur as presentations at national and international scientific meetings (e.g., the International Association of Providers of AIDS Care and the International AIDS Society meetings), publications in peer-reviewed journals (e.g., *AIDS and Behavior*), and presentations to public health entities (e.g., Romanian Ministry of Health). This clinical trial has been registered at *ClinicalTrials.gov*, to which information will be submitted in a timely manner. Results information will be submitted no later than 1 year after the trial's primary completion date, approximately August 2024. Research resources will be freely distributed, as available, to qualified academic investigators for non-commercial research. The PIs will adhere to the NIH Grants Policy on Sharing of Unique Research Resources including the “Sharing of Biomedical Research Resources: Principles and Guidelines for Recipients of NIH Grants and Contracts.” http://ott.od.nih.gov/policy/rt_guide_final.html.

## Abbreviations

AE: Adverse event
ARAS: Asociaţia Română Anti-SIDA (Romanian Association Against AIDS)
ASSIST: Alcohol, Smoking and Substance Involvement Screening Test
CEE: Central-Eastern Europe
CREM Cluj: Regional Center of Evidence and Monitoring of HIV Infection Cluj-Napoca
DBS: Dried blood spot
DSMB: Data and Safety Monitoring Board
EC: Education control
EU/EEA: European Union/European Economic Area
FDA: U.S. Food and Drug Administration
GAD-7: Generalized Anxiety Disorder 7-item
GBMSM: Gay, bisexual, and other men who have sex with men
HIPAA: Health Insurance Portability and Accountability Act
HMP: HealthMpowerment
ID: Identification number
IRB: Institutional Review Board
LGBTQ+: Lesbian, gay, bisexual, transgender and other identities
mHealth: Mobile health
NIH: U.S. National Institutes of Health
NIID: National Institute of Infectious Diseases
PHQ-9: Patient Health Questionnaire-9 item
PI: Principal investigator
PrEP: Pre-exposure prophylaxis
RCT: Randomized controlled trial
SPARK: Sparking PrEP Awareness Research and Knowledge
STI: Sexually transmitted infection
TDF/FTC: Tenofovir/emtricitabine
TFVdp: Tenofovir-diphosphate
U.S.: United States
WHO: World Health Organization
