# Correction: Prepare Romania: study protocol for a randomized controlled trial of an intervention to promote pre-exposure prophylaxis adherence and persistence among gay, bisexual, and other men who have sex with men

**DOI:** 10.1186/s13063-025-09106-z

**Published:** 2025-09-01

**Authors:** Corina Lelutiu-Weinberger, Mircea L. Filimon, Anna M. Zavodszky, Mihai Lixandru, Lucian Hanu, Cristina Fierbinteanu, Raluca Patrascu, Adrian Streinu-Cercel, Sergiu Luculescu, Maria Bora, Irina Filipescu, Cristian Jianu, Lisa B. Hightow-Weidman, Aimee Rochelle, Brian Yi, Nickie Buckner, Sarit A. Golub, Ilana Seager van Dyk, Julian Burger, Fan Li, John E. Pachankis

**Affiliations:** 1 School of Nursing, Columbia University, 560 West 168th Street, New York, NY 10032 USA; 2 The Romanian Association Against AIDS, Bulevardul Eroilor Sanitari 49, Bucharest, 050471 Romania; 3 The National Institute of Infectious Diseases “Professor Dr. Matei Bals”, Strada Doctor Calistrat Grozovici 1, Bucharest, 021105 Romania; 4 The Clinical Hospital of Infectious Diseases, Str. Iuliu Moldovan, nr. 23, Cluj-Napoca, 400000 Romania; 5 College of Nursing, Florida State University, 98 Varsity Way, Tallahassee, FL 32305 USA; 6 One Cow Standing, 300 W Morgan St Ste 1425, Durham, NC 27701 USA; 7 Department of Psychology, Hunter College of the City University of New York, 695 Park Avenue, New York, NY 10065 USA; 8 School of Psychology, Massey University, PO Box 756, Wellington, 6140 New Zealand; 9 Department of Social and Behavioral Sciences, Yale School of Public Health, 60 College Street, New Haven, CT 06610 USA; 10 Department of Biostatistics, Yale School of Public Health, 60 College Street, New Haven, CT 06520 USA; 11 Social and Behavioral Sciences, Yale School of Public Health, 135 College Street, New Haven, CT 06520 USA

**Correction: Trials 25, 470 (2024)**

**https://doi.org/10.1186/s13063-024-08313-4**

Following the publication of the original article [1], we were notified that an extra “e” was mistakenly added to the 13th author’s name.

Originally published name: Lisa B. Heightow-Weidman.

Correct name: Lisa B. Hightow-Weidman.

The original article has been corrected.
